# Treatment of Thyroid Dysfunctions Decreases the Risk of Cerebrovascular Events in Men but Not in Women: Results of the MONICA/KORA Cohort Study

**DOI:** 10.1371/journal.pone.0155499

**Published:** 2016-05-18

**Authors:** Julia Six-Merker, Christa Meisinger, Carolin Jourdan, Margit Heier, Hans Hauner, Annette Peters, Jakob Linseisen

**Affiliations:** 1 Institute of Epidemiology II, Helmholtz Zentrum München, German Research Center for Environmental Health (GmbH), Neuherberg, Germany; 2 MONICA/KORA Myocardial Infarction Registry, Central Hospital of Augsburg, Augsburg, Germany; 3 Else Kröner-Fresenius-Center for Nutritional Medicine, Klinikum rechts der Isar, Technical University of Munich, Munich, Germany; Hospital Universitario LA FE, SPAIN

## Abstract

**Objective:**

Thyroid disorders are well known to be associated with cardiovascular diseases. Some studies have shown that the negative effects of thyroid disorders are partially reversible after adequate treatment. The aim of this analysis was to assess the risk of incident ischemic cerebrovascular diseases in study participants treated for thyroid dysfunctions in a population-based cohort study.

**Methods:**

For the presented analyses data from 8564 male and 8714 female individuals aged 25 to 74 years of the MONICA/KORA cohort were used (median follow-up 14.0 years). A combined binary variable “thyroid disorder” (TDC) was created utilizing data on self-reported physician-treated thyroid disorders and information about medication use. To examine the association between TDC and incident ischemic cerebrovascular events, we performed multiple adjusted Cox proportional hazard regression models and calculated hazard ratios and corresponding 95% confidence intervals (HR, 95%CI).

**Results:**

During follow-up between 1984 and 2008/2009, 514 incident fatal and non-fatal ischemic cerebrovascular events occurred in men and 323 in women. At baseline, 3.5% of men and 15.6% of women reported TDC. In the fully adjusted model, males who reported TDC had a significantly reduced risk of ischemic cerebrovascular events (HR = 0.52, 95%CI = 0.29–0.92). A similar result was obtained in men, when we utilized information on thyroid hormones use only. For the total study population and for women with TDC we found no association with ischemic cerebrovascular events.

**Conclusions:**

In our longitudinal analyses subjects with treated thyroid diseases had no increased risk of incident ischemic cerebrovascular events. Surprisingly in males, even a significantly reduced risk of incident ischemic cerebrovascular events was found, a result that deserves further clarification.

## Introduction

Thyroid disorders are common diseases worldwide [1] and are known to be associated with several cardiovascular disorders [2–4]. The prevalence of thyroid diseases depends on age, gender, geographical factors, and iodine intake [5]. Hypothyroidism is the most frequent pathological condition with abnormally elevated thyroid stimulating hormone (TSH) levels; depending on the plasma concentration of thyroid hormones a distinction can be made between overt or subclinical hypothyroidism [6, 7]. In patients with hyperthyroidism tissues are exposed to TSH concentrations below the reference range in the subclinical as well as overt status; the overt status is additionally characterized by elevated production of thyroid hormones [6].

Cerebrovascular diseases are still one of the major causes of mortality worldwide [8] and they go along with many years of life lost and years lived with disabilities [9, 10]. Rather limited information exists about the association between thyroid disorders and incident cerebrovascular diseases [11–14], whereas several studies predominantly indicate a positive association between thyroid disorders and an increased risk of ischemic heart disease [3, 14–16]. However, little comparable information is available on whether this holds true for cerebrovascular disease as well [12].

Looking at studies with participants suffering from thyroid disorders and only partly treated, a more recent prospective study reported that hyperthyroidism was associated with an increased risk of stroke [7] whereas two larger studies reported no association between hyperthyroidism and stroke incidence [17, 18]. For participants of the First National Health and Nutrition Survey suffering from hypothyroidism an elevated risk for all strokes and ischemic stroke was observed [18]. Contrary to these results, a registry-based study was unable to detect a relation between hypothyroidism and stroke [17].

With the focus only on patients treated for thyroid disorders, a study based on clinical registry data could not demonstrate a relation between hyperthyroidism and cerebrovascular disease, but reported an increased risk of cerebrovascular disease in patients treated for hypothyroidism compared to the general population [14].

Thus, the information about the association between thyroid disorders and cerebrovascular diseases is limited and conflicting. The aim of this study, therefore, was to assess the risk of incident ischemic cerebrovascular diseases in study participants with thyroid dysfunctions in a prospective population-based study, comparing treated participants vs. non-treated persons from the general population.

## Materials and Methods

### Study design and participants

The presented analyses are based on the MONICA (MONitoring of Trends and Determinants in CArdiovascular Disease)/KORA (Cooperative Health Research in the Region of Augsburg) studies. At baseline, four independent study populations were recruited based on a random sample (1984/85 (S1), 1989/90 (S2), 1994/95 (S3), and 1999/2001 (S4) in the city of Augsburg and two surrounding counties with the aim to examine the distribution and time trends of cardiovascular risk factors [19, 20]. The response decreased gradually over the years from 79% in S1 to 65% in S4 [21, 22]. The age range of the participants was between 25 and 64 years at study entry in S1 and between 25 and 74 years in S2-S4. Subjects from all four surveys were prospectively followed up within the KORA framework until 2008/2009 [19, 23]. May 12, 2015). Of the initially included 17604 (8769 male, 8835 female) individuals in our dataset 326 persons were excluded due to a history of (any type of) stroke (n = 209) at baseline and/or an incident hemorrhagic cerebrovascular event during the follow-up (n = 129).

In all surveys, extensive baseline information was collected. More detailed data about study design, recruitment methods and data collection have been published previously [19].

Approval from the local ethics committee (Bayerische Landesärztekammer) was received. In accordance with institutional requirements and the Declaration of Helsinki all participants provided written informed consent after full explanation of the purpose and nature of all procedures used.

### Data collection procedures

Baseline status and risk factors such as demographic and sociodemographic variables, lifestyle factors, medication use and medical history were collected through standardized computer assisted interviews of participants by trained medical staff. In addition, participants underwent extensive physical examinations and health status assessment including body height and body weight. Furthermore, non-fasting blood samples were drawn while sitting. Details about the measurement procedures were described elsewhere [19, 20].

### Measures

#### Thyroid disorders

In epidemiologic studies, different information sources can be used to characterize subjects as euthyroid or suffering from thyroid dysfunction. The analysis of serum levels of TSH and thyroxine (T4) is frequently used to assess the thyroid function. A non-invasive option is to ask people about their medical diagnoses or assess and evaluate their medication use. Following this approach, it is possible to assess data about the longer-term health status and take into account current treatment. In our analyses, multiple non-invasive information sources were used to define four variables as proxies for treated thyroid disorders. During the medical face-to-face interview questions were asked if the study participants suffered from physician-treated thyroid disorders during the last 12 months and if they received treatment. In addition to the medical questions, all medications taken by the participant during the past 7 days were recorded including dosage and administration form. Utilizing the Anatomical Therapeutic Chemical (ATC) classification system from the WHO Collaborating Center for Drug Statistics Methodology allows a comparison of drug consumption at international level [24] and avoids false positive disease reports. The use of thyroid hormones is a common therapy and a clear indication of a current thyroid disorder, such as hypothyroidism and struma [25, 26]. Furthermore, they are prescribed regularly after struma/thyroidectomy [25, 26]. Beside radioactive iodine and surgery, thyreostatic medication is one of the main treatment options for hyperthyroidism [25–27]. The type of treatment depends on factors such as cause and severity of the disease, age of the patients, and the presence of coexisting conditions [25–27]. We utilized the questions about treated thyroid disorders in the last 12 months and also the information about thyroid hormone and thyreostatic medication use to create a combined binary variable “thyroid disorder” (TDC) (yes, no). Based solely on information from the medical anamnesis at baseline, the binary questionnaire variable “thyroid disorder” (TDQ) (yes, no) was generated. This variable does not distinguish between hypothyroidism and hyperthyroidism. With the information on thyroid hormone use only we created the variable “hypothyroidism/struma” (HOT) (yes, no) and with the information on thyreostatic medication use the variable “hyperthyroidism” (HET) (yes, no). HET could not be used for statistical tests or multivariate analyses due to the low number of cases.

#### Covariates

Information about age, sex, education (< vs. > = 12 years school and professional education), smoking habits (never, former/irregular current, regular current), physical activity (active, inactive), and alcohol consumption (g/day) was collected through standardized computer assisted interviews and additional questionnaires. Body mass index (BMI) was calculated as weight in kilograms divided by squared height in meters. The participants completed a self-administered, short food frequency questionnaire from which a healthy nutrition index was built (healthy, normal, unhealthy dietary pattern).

Hypertension, myocardial infarction, diabetes mellitus, hyperuricemia, and an unfavorable blood lipid ratio were included as covariates as they have been shown to be risk factors for cardiovascular diseases including cerebrovascular diseases [27–30]. Arterial hypertension (yes, no) was defined as a systolic blood pressure of >140mm Hg, and/or diastolic blood pressure of >90mm Hg, and/or current use of antihypertensive medication, given that the persons were aware of being hypertensive. Patient’s self-report about “in hospital treated myocardial infarctions” was used for the classification of myocardial infarction (yes, no). Diabetes mellitus was defined by the patient’s self-report of a physician diagnosed diabetes mellitus and/or current use of antidiabetic medications [31]. Hyperuricemia was defined as blood concentrations of uric acid exceeding 6.5 mg/dl [32]. For the statistical analyses, we computed the unfavorable lipid ratio (total cholesterol/HDL-cholesterol) because it is a simple and powerful predictor for coronary heart disease risk [33]. Ratios greater than 6 indicate a high risk for the onset of cardiovascular diseases [34, 35]. Additionally, for women menopause status was assessed during the interview and classified into three categories (postmenopausal, hormone therapy or menstruation, premenopausal).

#### Outcome and follow up

The end point used in these analyses were incident fatal and non-fatal ischemic cerebrovascular events (ICE) which include ischemic and embolic strokes, transient ischemic attacks “TIA”, prolonged ischemic neurological deficit “PRIND”, and further forms of ICE. These events were identified through follow-up activities which included regular checking of the subject’s vital status (through population registries inside and outside the study area), three postal questionnaires focusing on chronic diseases, and also follow-up studies in which the participants were invited to the KORA study center for medical examination and a standardized interview. An ICE was defined as incident if its occurrence was observed for the first time during the follow-up. Diagnoses were validated by autopsy reports, death certificates, clinical records, and information from the last treating physician. The underlying cause of death was coded using the ninth revision of the International Classification of diseases (ICD-9).

#### Statistical analyses

Baseline characteristics and additional variables, selected through literature research, were examined as median and interquartile range for continuous variables (variables did not meet the normality assumption) and as absolute and relative frequencies for categorical variables stratified by sex and the variables TDC and HOT. Bivariate tests for differences between participants with and without treated thyroid disorders were performed applying Wilcoxon-Mann-Whitney-tests for continuous parameters and Chi^2^ tests for categorical variables to check the statistical significance. With the selected variables out of the bivariate tests we examined the association between the three thyroid disorder variables TDC, TDQ, as well as HOT and ICE. Cox proportional hazard regression models with days as the timescale and calculation of hazard ratios (HR) and corresponding 95% confidence intervals (95%CI) were performed. Time at entry was the day at baseline examination. Exit time was the day when either a participant was diagnosed with an ICE, died, was lost to follow-up, or was censored at the end of the follow-up period. The crude model adjusted for age, sex, and survey was further adjusted in groups for lifestyle factors (education, BMI, alcohol consumption, smoking habits, physical activity, and dietary patterns), and clinical risk factors (history of arterial hypertension, diabetes mellitus, hyperuricemia, and unfavorable blood cholesterol ratio) until a fully adjusted model (crude model adjusted for lifestyle factors and clinical risk factors) was obtained. Additionally, the variable myocardial infarction (yes, no) in all participants and menopause status at baseline in women only were tested as adjusting variable. However, both did not change the risk estimates (data not shown) and were not included in the fully adjusted models.

To test the robustness of our models, we calculated the Cox proportional hazard regressions without embolic strokes as part of the outcome variables. Therefore, in this sensitivity analysis only incident ischemic strokes, TIAs/PRINDs and further undefined forms of ICE were included as relevant outcomes. Furthermore, we tested for interactions between the thyroid disorder variables (TDC, TDQ, as well as HOT) and sex, hypertension as well as alcohol consumption. Additionally, in women interaction between thyroid disorders and menopause status was assessed. To minimize the likelihood of reverse causal associations, we imposed a two-year lag period between baseline data ascertainment and the onset of observation time for ICE. All analyzed tests and regression models were also done stratified by gender.

Significance tests were 2-tailed. A p-value of less than 0.05 was considered significant. The assumption of proportionality of hazards was assessed by visualizing the log(-log(survival) plots of models stratified by risk factor categories. All interaction analyses were conducted by including a cross-product term along with the main effect terms in the Cox regression models. The statistical significance of the cross-product term was evaluated using the likelihood ratio test between models. The log-rank test was used to test for differences in the survivor function. For all analyses the software package SAS 9.3 (SAS Institute Inc., Cary, NC, USA) was used.

## Results

In the 25- to 74-year old study population (N = 17,278) at baseline 837 ICE were identified during the follow-up between 1984 and 2008/2009 (median follow-up time 14.0 years). In men 514 (median follow-up 13.9 years) and in women 323 (median follow-up 14.1 years) incident ICE were registered. From all incident ischemic cerebrovascular cases 167 were fatal, 89 among men and 78 among women.

At the baseline examination, significantly more women than men reported a treated thyroid disorder. This applies to the questions during the medical interview as well as thyroid hormone use or antithyroid medication use (men: TDC 3.5%, TDQ 2.9%, HOT 2.8%, HET 0.1%, women: TDC 15.6%, TDQ 13.6%, HOT 12.3%, HET 0.4%).

The baseline characteristics stratified by sex and TDC or HOT are described in Tables 1 and 2. Similar distributions were found among the different thyroid disorder categories (TDC, TDQ, HOT). Males and females treated for thyroid disorders were at baseline significantly older than non-treated persons. Furthermore, hypertension and diabetes mellitus were more common in men and women with treated thyroid disorders. The prevalence of myocardial infarction was independent from the presence of treated thyroid disorders. Women with treated thyroid disorders had a significantly higher BMI, a lower education and more often a diagnosis of hyperuricemia and they were more often in a postmenopausal status. Men suffering from thyroid disorders (treated) reported to smoke less regularly or never and to eat a diet with a healthier dietary pattern.

**Table 1:** Demographic, lifestyle and clinical factors by treated thyroid disorders (TDC) in men and women, KORA population S1-S4.

		Men				P-value	Women				P-value
		TDC yes		TDC no			TDC yes		TDC no		
Baseline characteristics		n = 302	%	n = 8259	%		n = 1359	%	n = 7353	%	
Age at baseline examination^a^		54.0	43.0–63.0	48.0	36.0–59.0	<0.0001^e^	51.0	42.0–61.0	47.0	36.0–59.0	<0.0001^e^
Survey	1	48	15.9	1938	23.5	0.0106^f^	267	19.6	1707	23.2	<0.0001^f^
	2	81	26.8	2255	27.3		335	24.6	2019	27.5	
	3	89	29.5	2153	26.1		353	26.0	1923	26.2	
	4	84	27.8	1913	23.2		404	29.7	1704	23.2	
**Lifestyle factors**											
Body mass index^a^	kg/m^2^	27.2	25.3–29.3	26.8	24.7–29.2	0.0764^e^	26.5	23.6–30.4	25.4	22.6–29.1	<0.0001^e^
Alcohol consumption^a^	g/day	20.0	2.9–38.0	20.4	5.7–42.9	0.0344^e^	2.8	0.0–11.7	2.8	0.0–14.3	0.0076^e^
Education	<12 years	189	62.6	5181	62.9	0.8982^f^	1115	82.1	5626	76.6	<0.0001^f^
	≥12 years	113	37.4	3050	37.0		243	17.9	1715	23.4	
Smoking habits	Regular current	61	20.2	2424	29.4	0.0005^f^	253	18.6	1366	18.6	0.6837^f^
	Former/ unregular	152	50.3	3352	40.7		315	23.2	1628	22.2	
	Never	89	29.5	2458	29.8		790	58.2	4350	59.2	
Physical activity	Active	118	39.1	3706	45.0	0.0402^f^	538	39.6	2981	40.6	0.4873^f^
	Inactive	184	60.9	4520	54.9		820	60.4	4357	59.3	
Dietary pattern	Unhealthy	111	36.8	3575	43.4	0.0012^f^	328	24.2	1970	26.8	0.1223^f^
	Normal	55	18.2	1785	21.7		291	21.4	1521	20.7	
	Healthy	136	45.0	2861	34.8		737	54.3	3843	52.3	
Menopause status	Postmenopausal						769	56.6	3356	45.7	<0.0001^f^
	Hormone intake and menstruation						31	2.3	163	2.2	
	Premenopausal						528	38.9	3684	50.2	
**Clinical factors/diseases**											
Hypertension	Yes	149	49.3	3583	43.5	0.0455^f^	519	38.2	2232	30.4	<0.0001^f^
	No	153	50.7	4649	56.5		838	61.7	5110	69.6	
Diabetes mellitus	Yes	20	6.6	332	4.0	0.0262^f^	76	5.6	224	3.0	<0.0001^f^
	No	282	93.4	7902	96		1282	94.4	7119	96.9	
Unfavorable lipid ratio^b^	Yes	63	20.9	1875	22.8	0.4315^f^	121	8.9	521	7.1	0.0200^f^
	No	234	77.5	6217	75.5		1203	88.6	6620	90.1	
Hyperuricemia^c^	Yes	77	25.5	2155	26.2	0.8427^f^	63	4.6	227	3.1	0.0040^f^
	No	218	72.2	5940	72.1		1264	93.1	6904	94.0	
Myocardial infarction	Yes	10	3.3	234	2.8	0.6307^f^	12	0.9	48	0.6	0.3466^f^
	No	292	96.7	8000	97.2		1346	99.1	7296	99.3	
**Additional variables**											
Thyreostatic medication	Yes	9	3.0	0	0.0		38	2.8	0	0.0	
	No	293	97.0	8234	100.0		1320	97.2	7345	100.0	
Thyroid hormone use	Yes	242	80.1	0	0.0		1068	78.6	0	0.0	
	No	60	19.9	8245	100.0		290	21.4	7350	100.0	
ischemic cerebrovascular events (total)^d^	Yes	14	4.6	498	6.0		52	3.8	270	3.7	
	No	276	91.4	7259	88.2		1208	89	6591	89.7	
embolic strokes	Yes	3	1.0	82	1.0		9	0.7	57	0.8	
	No	299	99	8152	99.0		1349	99.3	7288	99.2	
TIA/PRIND	Yes	0	0.0	122	1.5		12	0.9	61	0.8	
	No	290	96	7635	92.7		1248	91.9	6800	92.6	
Follow-up time^a^	Days	5004.5	3202.0–6882.0	5084.0	3209.0–6953.0		5027.0	3212.0–6976.5	5149.0	3305.0–7002.0	

TDC, treated thyroid disorders (yes/no) based on the information on self-reported physician-treated disease and medication use; TIA/PRIND, Transient ischaemic attack/prolonged ischaemic neurological deficit. Data are presented in n (%) or median (interquartile range).

^a^ Median und Interquartile range.

^b^ Total cholesterol (mg/dl)/high-density lipoprotein (mg/dl), ≥6 (yes), <6 (no).

^c^ Uric acid ≥ 6.5 mg/dl.

^d^ Include ischemic and embolic strokes, transient ischaemic attacks “TIA”, prolonged ischaemic neurological deficit “PRIND”, and further forms of ischemic cerebrovascular events.

^e^ Wilcoxon-Mann-Whitney test.

^f^ Chi^2^ test.

**Table 2:** Demographic, lifestyle and clinical factors by thyroid hormone use (HOT) in men and women, KORA population S1-S4.

		Men				P-value	Women				P-value
		HOT yes		HOT no			HOT yes		HOT no		
Baseline characteristics		n = 242	%	n = 8305	%		n = 1068	%	n = 7640	%	
Age at baseline examination^a^		55.5	43.0–63.0	48	36.0–59.0	<0.0001^e^	52.0	43.0–61.0	47.0	36.0–59.0	<0.0001^e^
Survey	1	40	16.5	1943	23.4	0.0595^f^	204	19.1	1768	23.1	0.0007^f^
	2	69	28.5	2262	27.2		283	26.5	2071	27.1	
	3	65	26.8	2177	26.2		275	25.8	2001	26.2	
	4	68	28.1	1923	23.2		306	28.6	1800	23.6	
**Lifestyle factors**											
Body mass index^a^	kg/m^2^	27.2	25.3–29.3	26.8	24.7–29.2	0.0871^e^	26.7	23.9–30.7	25.4	22.6–29.1	<0.0001^e^
Alcohol consumption^a^	g/day	20.0	4.4–37.1	20.4	5.7–42.9	0.0538^e^	2.8	0.0–11.4	2.8	0.0–14.3	0.0079^e^
Education	<12 years	152	62.8	5225	62.9	0.9641^f^	885	82.9	5857	76.7	<0.0001^f^
	≥12 years	90	37.2	3075	37.0		183	17.1	1776	23.2	
Smoking habits	Regular current	48	19.8	2441	29.4	0.0003^f^	186	17.4	1434	18.8	0.5563^f^
	Former/ unregular	128	53.9	3380	40.7		244	22.8	1699	22.2	
	Never	66	27.3	2483	29.9		638	59.7	4505	59.0	
Physical activity	Active	97	40.1	3728	44.9	0.1314^f^	423	39.6	3096	40.5	0.5409^f^
	Inactive	145	59.9	4561	54.9		645	60.4	4532	59.3	
Dietary pattern	Unhealthy	88	36.4	3600	43.4	0.0007^f^	241	22.6	2057	26.9	0.0095^f^
	Normal	41	16.9	1800	21.7		238	22.3	1574	20.6	
	Healthy	113	46.7	2884	34.7		587	55.0	3993	52.3	
Menopause status	Postmenopausal						633	59.3	3492	45.7	<0.0001^f^
	Hormone intake and menstruation						24	2.2	170	2.2	
	Premenopausal						390	36.5	3822	50.0	
**Clinical factors/ diseases**											
Hypertension	Yes	125	51.6	3613	43.5	0.0120^f^	421	39.4	2331	30.5	<0.0001^f^
	No	117	48.4	4687	56.4		646	60.5	5302	69.4	
Diabetes mellitus	Yes	16	6.6	337	4.1	0.0491^f^	68	6.4	232	3.0	<0.0001^f^
	No	226	93.4	7967	95.9		1000	93.6	7406	96.9	
Unfavorable lipid ratio^b^	yes	48	19.8	1893	22.8	0.2752^f^	95	8.9	548	7.2	0.0460^f^
	No	190	78.5	6269	75.5		946	88.6	6881	90.1	
Hyperuricemia^c^	Yes	64	26.4	2169	26.1	0.8808^f^	54	5.1	237	3.1	0.0010^f^
	No	173	71.5	5995	72.2		988	92.5	7184	94.0	
Myocardial infarction	Yes	9	3.7	235	2.8	0.4134^f^	5	0.5	55	0.7	0.3507^f^
	No	233	96.3	8067	97.13		1063	99.53	7579	99.2	
**Additional variables**											
Thyreostatic medication	Yes	3	1.2	6	0.1		12	1.1	26	0.3	
	No	239	98.8	8299	99.9		1056	98.9	7614	99.7	
Ischemic cerebrovascular events (total)^d^	Yes	12	5.0	500	6.0		41	3.8	2.81	3.7	
	No	221	91.3	7325	88.2		952	89.1	6851	89.7	
Embolic strokes	Yes	3	1.2	82	1.0		9	0.8	57	0.8	
	No	239	98.8	8223	99.0		1059	99.2	7583	99.3	
TIA/PRIND^e^	Yes	0	0.0	122	1.4		10	0.9	63	0.8	
	No	233	96.3	7703	92.8		983	92.0	7069	92.5	
Follow-up time^a^	Days	5034.0	3214.0–6908.0	5082.0	3207.0–6951.0		5057.0	3227.0–6986.0	5141.0	3299.5–6999.0	

HOT, hypothyroidism/struma (yes, no) classification based on thyroid hormone use; TIA/PRIND, Transient ischaemic attack/prolonged ischaemic neurological deficit.

Data are presented in n (%) or median (interquartile range).

^a^ Median und Interquartile range.

^b^ Total cholesterol (mg/dl)/high-density lipoprotein (mg/dl), ≥6 (yes), <6 (no).

^c^ Uric acid ≥ 6.5 mg/dl

^d^ Include ischemic and embolic strokes, transient ischaemic attacks “TIA”, prolonged ischaemic neurological deficit “PRIND”, and further forms of ischemic cerebrovascular events.

^e^ Wilcoxon-Mann-Whitney test.

^f^ Chi^2^ test.

Among the 9 hyperthyroid men in our study population no incident ICE occurred during follow-up; among the 38 hyperthyroid women two incident ICE were diagnosed.

The hazard ratios and corresponding 95% confidence intervals for the association of treated thyroid disorder (TDC, TDQ or HOT) and ICE are given in Table 3. The results for all three thyroid disorder variables were quite comparable. Considering the entire study population, in the crude model, in the model adjusted for lifestyle factors as well as in the fully adjusted model, study participants who reported a treated thyroid disorder had no change in risk for an incident ICE in comparison to study participants without a treated thyroid disorder (fully adjusted model: TDC, HR = 0.80, 95%CI = 0.61–1.04; TDQ, HR = 0.87, 95%CI = 0.65–1.15; HOT, HR = 0.75, 95%CI = 0.56–1.01).

**Table 3:** Gender specific and overall hazard ratios (HR) and 95%CI for developing an ischemic cerebrovascular event according to thyroid disorders at baseline.

		Crude model^a^		Lifestyle model^b^		Lifestyle and clinical risk factor model^c^	
		HR	(95%CI)	HR	(95%CI)	HR	(95%CI)
**TDC**	n^e^						
**Total**^**d**^	15,611	0.84	(0.65–1.09)	0.83	(0.64–1.07)	0.80	(0.61–1.04)
**Men**	7,831	0.58	(0.34–0.99)	0.55	(0.31–0.95)	0.52	(0.29–0.92)
**Women**	7,780	1.00	(0.74–1.34)	0.98	(0.73–1.33)	0.94	(0.69–1.28)
**TDQ**							
**Total**^**d**^	15,608	0.90	(0.69–1.18)	0.88	(0.67–1.16)	0.87	(0.65–1.15)
**Men**	7,828	0.70	(0.41–1.20)	0.67	(0.38–1.16)	0.66	(0.37–1.17)
**Women**	7,780	1.03	(0.75–1.41)	1.01	(0.73–1.39)	0.97	(0.70–1.35)
**HOT**							
**Total**^**d**^	15,604	0.80	(0.60–1.06)	0.78	(0.59–1.04)	0.75	(0.56–1.01)
**Men**	7,826	0.59	(0.33–1.05)	0.55	(0.30–0.99)	0.49	(0.26–0.92)
**Women**	7,778	0.92	(0.66–1.28)	0.91	(0.65–1.28)	0.88	(0.62–1.23)

HR, Hazard ratio; CI, Confidence interval; TDC, thyroid disorders (yes, no) classification based on a combination of medical questionnaire and medication use; TDQ, thyroid disorders (yes, no) classification questionnaire based; HOT, hypothyroidism/struma (yes, no) classification based on thyroid hormone use.

^a^ adjusted for age and survey.

^b^ adjusted for age, survey and lifestyle factors: education, smoking habits, physical activity, alcohol consumption, BMI, dietary pattern.

^c^ adjusted for age, survey, lifestyle factors and clinical risk factors (hypertension, diabetes mellitus, hyperuricemia and unfavorable blood cholesterol ratio).

^d^ additionally adjusted for sex in all models.

^e^ n from lifestyle and clinical risk factor model.

For men suffering from thyroid disorders (treated) the crude model (adjusted for age and survey) showed a significantly reduced risk of ICE compared to men without treated thyroid disorders. This association remained stable in the fully adjusted model. In two of the three thyroid disorder categories the results reached the significance level (fully adjusted model: TDC, HR = 0.52, 95%CI = 0.29–0.92; TDQ, HR = 0.66, 95%CI = 0.37–1.17; HOT, HR = 0.49, 95%CI = 0.26–0.92).

We did not see any statistically significant effects in the same models for women (fully adjusted model: TDC, HR = 0.94, 95%CI = 0.37–1.17; TDQ, HR = 0.97, 95%CI = 0.37–1.17; HOT, HR = 0.88, 95%CI = 0.37–1.17).

As demonstrated in the sensitivity analyses, embolic strokes had no relevant impact on the association between treated thyroid disorders and the risk of ICE. The results are shown in Table 4. None of the p-values for interaction reached the significance level.

**Table 4:** Gender specific and overall hazard ratios (HR) and 95%CI for developing an ischemic cerebrovascular event (without embolic stroke) according to thyroid disorders at baseline.

		Crude model^a^		Lifestyle model^b^		Lifestyle and clinical risk factor model^c^	
		HR	(95%CI)	HR	(95%CI)	HR	(95%CI)
**TDC**	n^e^						
**Total**^**d**^	15,466	0.86	(0.65–1.14)	0.87	(0.65–1.15)	0.83	(0.62–1.11)
**Men**	7,749	0.54	(0.30–0.99)	0.55	(0.30–1.00)	0.51	(0.27–0.96)
**Women**	7,717	1.05	(0.76–1.46)	1.05	(0.75–1.46)	0.99	(0.71–1.40)
**TDQ**							
**Total**^**d**^	15,463	0.93	(0.70–1.25)	0.94	(0.70–1.26)	0.91	(0.67–1.24)
**Men**	7,746	0.65	(0.36–1.19)	0.67	(0.37–1.22)	0.65	(0.35–1.23)
**Women**	7,717	1.11	(0.78–1.56)	1.10	(0.78–1.55)	1.04	(0.73–1.48)
**HOT**							
**Total**^**d**^	15,460	0.77	(0.56–1.07)	0.78	(0.57–1.08)	0.74	(0.53–1.03)
**Men**	7,745	0.53	(0.27–1.02)	0.53	(0.27–1.03)	0.47	(0.23–0.94)
**Women**	7,715	0.92	(0.63–1.33)	0.92	(0.63–1.30)	0.88	(0.60–1.28)

HR, Hazard ratio; CI, Confidence interval; TDC, thyroid disorders (yes, no) classification based on a combination of medical questionnaire and medication use; TDQ, thyroid disorders (yes, no) classification questionnaire based; HOT, hypothyroidism/struma (yes, no) classification based on thyroid hormone use.

^a^ adjusted for age and survey.

^b^ adjusted for age, survey and lifestyle factors: education, smoking habits, physical activity, alcohol consumption, BMI, dietary pattern.

^c^ adjusted for age, survey, lifestyle factors and clinical risk factors (hypertension, diabetes mellitus, hyperuricemia and unfavorable blood cholesterol ratio).

^d^ additionally adjusted for sex in all models.

^e^ n from lifestyle and clinical risk factor model

## Discussion

The main findings of this population-based, prospective study with more than 17,000 adults was that study participants who suffered from treated thyroid disorder had no higher risk for incident ischemic cerebrovascular events (ICE). Furthermore, a protective effect of treated thyroid disorders, especially treated hypothyroidism, regarding incident ICE was seen in men, but not women. The findings were stable even after adjusting for lifestyle and clinical risk factors. However, to the best of our knowledge, no other study observed sex-differences regarding this issue with a reduced risk for ICE only in men treated for thyroid disorders.

The major part of studies investigating the association between thyroid disorders and cerebrovascular diseases used serum hormone concentrations (TSH and T4) for the classification into diseased or healthy participants. Additionally, some of the studies collected information about medication use or medical history and usually used this information to exclude participants suffering from thyroid disorders at baseline. Comparing our results (all study participants) with a recent meta-analysis adjusted for multiple confounders, they correspond to the findings [12]. This meta-analysis included five prospective cohort studies and provided no evidence for an increased risk of stroke associated with subclinical hypothyroidism [12]. Furthermore, in a Danish register-based retrospective cohort study no association between subclinical or overt hypothyroidism and stroke was reported [17]. Contrary to these results, Qureshi et al. found by examining the data from the 20-year follow-up of the First National Health and Nutrition Survey those participants with hypothyroidism had a higher relative risk of all strokes and of ischemic stroke [18]. In a recent meta-analysis including 17 prospective cohorts with individual participant data of 47573 adults an increased risk of stroke events was found among participants with subclinical hypothyroidism compared to euthyroidism in the age groups 18–49 and 50–64 years, but not among participants aged 65–79 years and > = 80 years [36]. In a Taiwanese study utilizing data from a hospital database and including 5793 hypothyroidism patients Yang et al. showed that hypothyroidism increased the risk for cerebrovascular diseases (HR 1.89) [37]. However, if only blood levels are used it has be considered that the classification (diseased versus healthy) was based on a single measurement, which itself depends on factors such as circadian rhythm of TSH [38], diagnostic TSH assay variation, respective TSH reference ranges [38], or drug intake (e.g. psychotropic drugs, thyroid hormones) [39].

Utilizing data on medical interview data and information on medication use we computed the variable thyroid disorder in three different ways: First by combination of interview data about prevalent treated thyroid disorders, antithyroid medication use and thyroid hormone use—TDC, second by medical anamnesis only—TDQ, and third by thyroid hormone use only–HOT. For both the combined variable and the thyroid hormone use variable a significantly reduced risk of ICE was seen in men only. To date, only a few studies exist, which focus on the association between treated thyroid disorders and cerebrovascular diseases and only one reported results comparable to ours. Contrary to our results, a Scottish study based on clinical registry data focusing on patients treated for thyroid disorders, reported an increased risk of cerebrovascular diseases in patients treated for hypothyroidism compared to the general population [14]. A reason for these differences could be that the Scottish study could not adjust for lifestyle factors such as smoking and BMI [14] which are associated with thyroid disorders and stroke [40–43].

Our finding of a significantly reduced risk of ICE only in men with treated thyroid disorders was unexpected. Beside gender differences in the etiology of hypothyroidism [44], one explanatory approach for the gender difference in our findings could be a gender-specific risk profile. For example Yonemoto et al. [45] reported, that BMI as well as a combination of obesity and diabetes were risk factors for ischemic stroke in men only.

Another possible explanation for the observed gender-specific association between treated thyroid disorder and ICE could be the tendency of males to delay medical care until an advanced or more noticeable stage of disease [46]. Thus, men not under treatment may more often have an advanced stage of disease when they go to the doctor, which may leads to rarer misdiagnosis and more often to adequate treatment. While our data may reflect regional pattern, the under-treatment in males, reported from other European regions [47] seems not to occur in our sample.

The strengths of our study are the prospective design, the large sample size drawn from the general population and the variety of available data on lifestyle factors, health status, and important clinical parameters, which allowed multiple adjustments to control for potential confounding. The consistency of the results in the sensitivity analyses was quite reassuring.

However, there are also limitations which should be noted. The MONICA/KORA Augsburg Cohort study was limited to men and women aged 25 to 74 years from the south of Germany, thus caution is needed in generalizing our results to people of other populations and age-ranges. Another limitation of our data was the self-reported nature of the medical history, without verification. As described in the publication from Brix et al. 2001 the validity of self-reported hypothyroidism and hyperthyroidism achieves a high sensitivity, but only moderate specificity due to the high number of false-positive reports [48]. The utilization of the ATC classification system avoids false positive disease reports. For this reason, we combined self-reported diagnoses with medication use based on the ATC-code drug classification to get a more reliable and not invasive information source. Nevertheless, the prevalences of thyroid dysfunctions in our cohort were comparable to the findings in the existing literature confirming our method. At baseline examination, the prevalences of thyroid disorders ascertained with medical interview questions and medication use (thyroid hormones and antithyroid medication) were about 3.5% in men and about 13.6% in women. In the existing literature the overall prevalence for all types of thyroid disorders in northern Europe including Germany collected via questionnaire and medication use ranges from 1.5 to 8.3% in men and from 7.9 to 18.0% in women[49]. With information from medication use the prevalence of HOT were 2.8% in men and 12.3% in women. The Robert Koch Institute/Germany published slightly lower prevalence rates for hypothyroidism/goiter based on medical history questions and thyroid hormone use (1.6% in men, 8.1% in women) [49]. Two studies in Germany using serum parameter observed prevalences of hypothyroidism depending on the study participant’s age between 2.2% and 13.9% in men and between 1.7% and 31.0% in women [50]. In the USA, large studies have found prevalences of overt hypothyroidism (based on serum parameter) varying from 0.2–1.4% and prevalences of subclinical hypothyroidism in the range of 3.8–13.9% for men and women combined [51–53]. Irrespective of the underlying data source and varying serum blood TSH and fT4 limits for classification, hypothyroidism is more common with advancing age and in women [1, 49, 52]. This is confirmed by our results.

The absence of serum thyroid hormone data is also a limitation our analyses.

Despite the fact that we adjusted for a variety of variables, adjustment may be insufficient to rule out residual confounding, potentially in men. Also competing risk could be an influencing factor in the analyses. After exclusion of participants who had an ICE in the first two years, no changes in the results were observed so that reverse causation could be ruled out. Thyroid disorders are more common in women and cerebrovascular events more common in men, which leads to a difference distribution of outcome and predictor variables between men and women and to limited statistical power in the former. In addition, we had no data available on atrial fibrillation for use as a co-variate and the number of individuals with hyperthyroidism was too small in our study for meaningful statistical analyses.

## Conclusion

In summary, we found that men from the general population treated for thyroid disorders had a significantly reduced risk of ischemic cerebrovascular events compared to men without thyroid disorders. For the whole study population and women with treated thyroid disorder we saw no association at all. These findings underline the importance of thyroid disorder treatment. However, in the available literature results vary and further research examining thyroid disorders and their treatment are requested. Further adequately powered studies using information on blood hormone data, physician-diagnosed diseases and medication use, would be helpful.
